# Predicting Antibiotic Effect of Vancomycin Using Pharmacokinetic/Pharmacodynamic Modeling and Simulation: Dense Sampling versus Sparse Sampling

**DOI:** 10.3390/antibiotics11060743

**Published:** 2022-05-31

**Authors:** Yong Kyun Kim, Jae Ha Lee, Hang-Jea Jang, Dae Young Zang, Dong-Hwan Lee

**Affiliations:** 1 Division of Infectious Diseases, Department of Internal Medicine, Hallym University Sacred Heart Hospital, Hallym University College of Medicine, Anyang 14066, Korea; amoureuxyk@hallym.or.kr; 2 Division of Pulmonology and Critical Care Medicine, Department of Internal Medicine, Inje University Haeundae Paik Hospital, Inje University College of Medicine, Busan 48108, Korea; anilleus@naver.com (J.H.L.); okabango21@gmail.com (H.-J.J.); 3 Division of Hematology-Oncology, Department of Internal Medicine, Hallym University Sacred Heart Hospital, Hallym University College of Medicine, Anyang 14066, Korea; fhdzang@gmail.com; 4 Department of Clinical Pharmacology, Hallym University Sacred Heart Hospital, Hallym University College of Medicine, Anyang 14066, Korea

**Keywords:** vancomycin, pharmacokinetics, structural model, number of compartments, pharmacokinetic/pharmacodynamic index, AUC/MIC, probability of target attain

## Abstract

This study aimed to investigate the effect of a structural pharmacokinetic (PK) model with fewer compartments developed following sparse sampling on the PK parameter estimation and the probability of target attainment (PTA) prediction of vancomycin. Two- and three-compartment PK models of vancomycin were used for the virtual concentration–time profile simulation. Datasets with reduced blood sampling times were generated to support a model with a lesser number of compartments. Monte Carlo simulation was conducted to evaluate the PTA. For the two-compartment PK profile, the total clearance (CL) of the reduced one-compartment model showed a relative bias (RBias) and relative root mean square error (RRMSE) over 90%. For the three-compartment PK profile, the CL of the reduced one-compartment model represented the largest RBias and RRMSE, while the steady-state volume of distribution of the reduced two-compartment model represented the largest absolute RBias and RRMSE. A lesser number of compartments corresponded to a lower predicted area under the concentration–time curve of vancomycin. The estimated PK parameters and predicted PK/PD index from models built with sparse sampling designs that cannot support the PK profile can be significantly inaccurate and unprecise. This might lead to the misprediction of the PTA and selection of improper dosage regimens when clinicians prescribe antibiotics.

## 1. Introduction

Vancomycin is a tricyclic glycopeptide antimicrobial that inhibits the biosynthesis of the peptidoglycan of the bacterial cell wall. Since vancomycin was isolated from *Amycolatopsis orientalis* in 1953 and introduced into the market in 1958, it remains a crucial therapeutic option for serious bacterial infections [1]. Vancomycin is effective against gram-positive bacteria that are resistant to other antimicrobials, especially methicillin-resistant *Staphylococcus aureus* (MRSA) and methicillin-resistant *Staphylococcus epidermidis* [2]. Common adverse effects of vancomycin treatment include nephrotoxicity, ototoxicity, and infusion-related toxicities. Notably, nephrotoxicity is a major concern with vancomycin treatment. According to a meta-analysis of 15 studies, the incidence of nephrotoxicity varied from 5 to 43% and occurred on average between 4 and 17 days after the start of the vancomycin treatment. The odds of nephrotoxicity were associated with high trough concentrations of >15 mg/L (odds ratio, 2.67) and the incidence of nephrotoxicity incrementally increased as a function of the exposure duration from 6 to 45% during 7 to 14 days [3]. A study of 22,057 patients in a veteran population found that acute kidney injury was more than quadrupled in patients with trough levels >20 mg/L compared to those with trough levels <10 mg/L [4].

When monitoring the appropriateness of antibiotic therapy and determining an optimal dosage regimen, the PK describing the time course of drug exposure or concentration and the pharmacodynamics (PD) explaining drug response are simultaneously considered. The PK/PD index for vancomycin therapy is the area under the concentration–time curve at a steady state over 24 h above the minimum inhibitory concentration (AUC/MIC) [5]. A latest consensus guideline by the American Society of Health-System Pharmacists, the Infectious Diseases Society of America, the Pediatric Infectious Diseases Society, and the Society of Infectious Diseases Pharmacists recommends utilizing AUC values estimated by Bayesian modeling techniques for vancomycin treatment monitoring in serious MRSA-infected patients to achieve clinical efficacy and minimize toxicity [6].

To build useful models, population PK studies of vancomycin are still ongoing in various patient groups considering the presence of extracorporeal oxygenation therapy or continuous renal replacement therapy as well as sex, age group (neonates, infants, children, adults, and elderly patients), weight, body mass index, fat-free mass, body surface area, renal function, and other demographic or pathophysiologic factors [7,8]. However, essentially all models are wrong, but some are useful [9]. When the predictive performance of six published PK models of vancomycin developed in ICU patients were evaluated using data from two large cohorts of ICU patients, only one model was able to accurately predict the PK profiles [10]. The reason for the poor predictive performance lies not only in the differences in patient characteristics and covariate effects but also in the misspecification in structural model building [11]. In a study reviewing 30 population PK studies of vancomycin in adult patients, two-compartment models were found to be the most common structural models (*n* = 14) followed by one-compartment models (*n* = 13) and three-compartment models (*n* = 3) [8]. The median number of samples per patient ranged from 1.71 to 3.2 for one-compartment models, from 1.89 to 12 for two-compartment models, and from 10.5 to 19.5 for three-compartment models [8]. A lesser number of samples per patient tend to correspond to a lesser number of compartments in the model. There is a possibility that a good model cannot be built with insufficient information to distinguish compartments. Models that do not describe actual PK profiles of vancomycin may make it difficult to adjust the AUC-guided optimal dosage regimen.

Thus, this study aimed to investigate the effect of a reduced structural model with fewer compartments developed following sparse sampling on the estimation of the PK parameters and the prediction of the PK/PD index of vancomycin.

## 2. Materials and Methods

### 2.1. Simulation of the Concentration–Time Profiles

**Figure 1:**
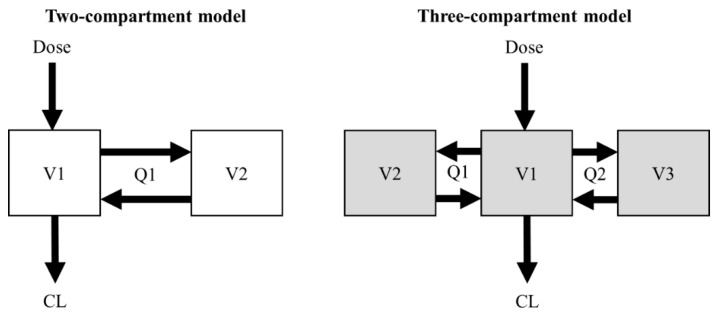
Structural design of the two- and three-compartment models.

Two- and three-compartment population PK models of vancomycin were used for the simulation of the virtual concentration–time profiles [12,13]. The structural PK parameters for the two-compartment model were total clearance (CL), central volume of distribution (V1), peripheral volume of distribution (V2), and intercompartmental clearance between V1 and V2 (Q1). The structural PK parameters for the three-compartment model were CL, V1, volume of distribution for the first peripheral compartment (V2), Q1, volume of distribution for the second peripheral compartment (V3), and intercompartmental clearance between V1 and V3 (Q2) (Figure 1).

The final PK parameter estimates of the initially developed two models used for simulation are described in Table 1. The structural PK parameters were assumed to follow log-normal distributions and were simulated by applying reported between-subject variability (BSV) within two standard deviations. The covariate values included in the original models were fixed to median values.

After generating the concentration–time profile according to the blood sampling times reported in the literature, a dataset with reduced blood sampling times was generated so that a model with a lesser number of compartments was inevitably developed. Based on the administration schemes of each study, the dosage regimens for simulation were 1 g over 2 h infusion for the two-compartment model and 1 g over 1 h infusion for the three-compartment model. A total of 200 datasets of concentration–time profiles were generated for each sample size of 12, 25, 50, and 100 (i.e., 12 PK profiles × 200, 25 PK profiles × 200, 50 PK profiles × 200, and 100 PK profiles × 200). The sampling times for the two-compartment PK profile simulation were 0, 2.5, 3, 4, 6, and 8 h. In this simulation data, the blood sampling times chosen to develop a PK model were 0, 2.5, 6, and 8 h for a one-compartment model and 0, 2.5, 3, 4, 6, and 8 h for a two-compartment model. The sampling times for the three-compartment PK profile simulation were 0, 1, 1.5, 2, 3, 4, 6, 8, and 12 h. In this simulation data, the blood sampling times chosen to develop a PK model were 0, 1.5, 6, and 12 h for a one-compartment model, 0, 1.5, 3, 4, 8, and 12 h for a two-compartment model, and 0, 1, 1.5, 2, 3, 4, 6, 8, and 12 h for a three-compartment model.

### 2.2. Estimation of PK Parameters

Each dataset was estimated using a first-order conditional estimation with an interaction method in a nonlinear mixed-effects modeling software (NONMEM^®^ 7.5, ICON Clinical Research LLC, North Wales, PA, USA). One-, two-, and three-compartment PK model building were performed using ADVAN1 TRANS2, ADVAN3 TRANS4, and ADVAN11 TRANS 4, respectively, from the model library of NONMEM. From 1600 datasets generated through the original two-compartment model, 200 one-compartment model parameter estimates of CL and V and 200 two-compartment model parameter estimates of CL, V1, Q1, and V2 were obtained for each sample size. From 2400 datasets generated through the original three-compartment model, 200 one-compartment model parameter estimates of CL and V; 200 two-compartment model parameter estimates of CL, V1, Q1, and V2; and 200 three-compartment model parameter estimates of CL, V1, Q1, V2, Q2, and V3 were obtained for each sample size group.

The bias and precision of the parameter estimates for the structural PK models were assessed using relative bias (RBias) and relative root mean square error (RRMSE) [14,15].

$$\mathrm{RBias}\;\left(\%\right)=100\times \frac{1}{N}\overset{N}{\underset{i}{\sum }}\frac{\theta_{i}-\theta_{T}}{\theta_{T}}$$

$$\mathrm{RRMSE}\;\left(\%\right)=100\times \sqrt{\frac{1}{N}\overset{N}{\underset{i}{\sum }}{\left(\frac{\theta_{i}-\theta_{T}}{\theta_{T}}\right)}^{2}}$$

where *θ_i_* is a PK parameter estimate and *θ_T_* is the parameter value used for PK profile simulation. The criteria for accuracy and precision were less than or equal to 15 and 35%, respectively [5].

### 2.3. Evaluation of the PK/PD Index of Vancomycin

The parameter estimates of the one- and two-compartment models established by fitting the PK profiles generated by the previously reported two-compartment model and those of the one-, two-, and three-compartment modes established by fitting the PK profiles generated by the previously reported three-compartment model were used for Monte Carlo simulation to evaluate the probability of meeting the PK/PD indices of AUC/MIC of <400, AUC/MIC of 400 to 600, and AUC/MIC of >600. A total of 10,000 PK parameters estimated using 200 simulated datasets with 50 subjects each were used for the Monte Carlo simulation to evaluate the probability of meeting the three PK/PD indices when MIC was 1 mg/L. The 200 generated datasets with 12, 25, and 100 subjects each were not used for this process. To explore the change in the probability of satisfying the PK/PD index with a change in doses, AUC at a steady state of over 24 h was calculated when 0.5, 0.75, and 1 g were given at 12 h intervals, respectively. The peak and trough concentrations at steady state were also investigated. R software (version 4.1.24, [www.rproject.org, accessed on 5 February 2022]) was used for postprocessing of model output and visualization.

## 3. Results

### 3.1. Estimation of PK Parameters

**Figure 2:**
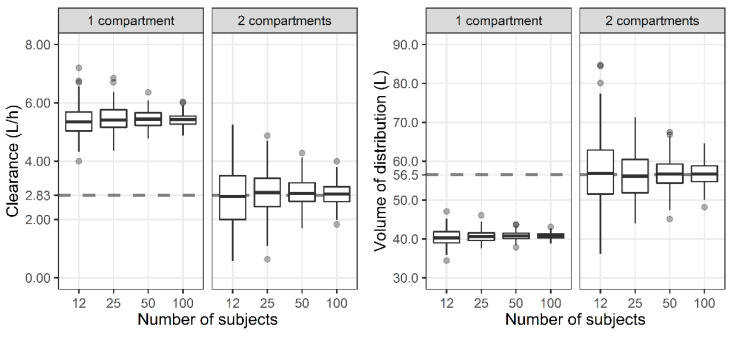
Distribution of total clearance and steady-state volume of distribution when one- and two-compartment models were developed with two-compartment PK profile and sparse or dense sampling designs (dashed lines, typical parameter values used for simulation; horizontal line inside the box, median; ends of each box, upper and lower quartiles; whiskers, 1.5 × quartiles; grey dots, outliers).

The CL and steady-state volume of distribution (V_SS_) of one-, two-, and three-compartment PK models were evaluated. The V_SS_ for one-, two-, and three-compartment models are V1, V1 + V2, and V1 + V2 + V3, respectively. For the two-compartment PK profile simulated using the initially developed two-compartment model, the reduced one-compartment model showed a larger CL and smaller V_SS_ compared with the two-compartment model (Figure 2). When the subject number was small, the distribution of the estimates was wide, while the median of the 200 estimated parameters was similar.

**Figure 3:**
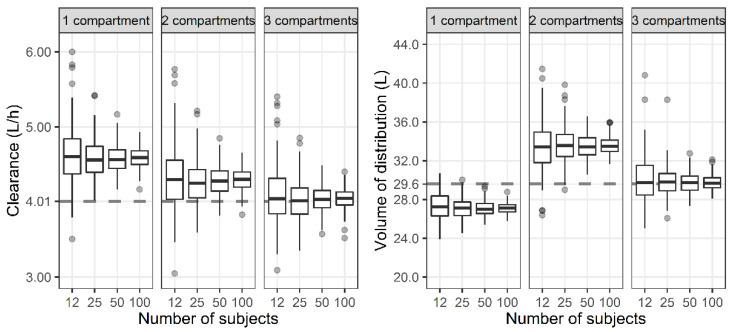
Distribution of total clearance and steady-state volume of distribution when one-, two-, and three-compartment models were developed with three-compartment PK profile and sparse or dense sampling designs (dashed lines, typical parameter values used for simulation; horizontal line inside the box, median; ends of each box, upper and lower quartiles; whiskers, 1.5 × quartiles; grey dots, outliers).

For the three-compartment PK profile, the reduced one- and two-compartment model showed a larger CL compared with the three-compartment model (Figure 3). In the case of the V_SS_, the one-compartment model represented smaller values, while the two-compartment model represented larger values compared with the three-compartment model.

### 3.2. Bias and Precision of PK Parameter Estimates

The accuracy and precision of the CL and V_SS_ were investigated using the RBias and RRMSE (Table 2). For the two-compartment PK profile, the CL of the reduced one-compartment model showed an RBias and RRMSE over 90%. The V_SS_ of the one-compartment model showed a negative RBias close to −30%. For the three-compartment PK profile, the CL of the one-compartment model represented the largest RBias and RRMSE compared to the other models, while the V_SS_ of the two-compartment model represented the largest absolute RBias and RRMSE. As expected, both the RBias and RRMSE tended to decrease with an increasing sample size.

### 3.3. Evaluation of the PK/PD Index of Vancomycin

**Figure 4:**
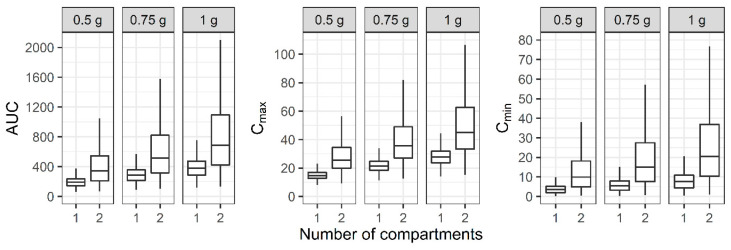
Distribution of area under the curve over 24 h (AUC), maximum concentration (C_max_), and minimum concentration (C_min_) at steady state when one- and two-compartment models were developed with two-compartment PK profile and sparse or dense sampling designs (dashed lines, typical parameter values used for simulation; horizontal line inside the box, median; ends of each box, upper and lower quartiles; whiskers, 1.5 × quartiles).

For the two-compartment PK profile, the AUC, C_max_, and C_min_ of the reduced one-compartment model showed smaller values compared to the two-compartment model (Figure 4). When a regimen of 0.75 g IV infusion over 2 h every 12 h was administered, the median AUC of the two-compartment model was between 400 and 600, while the median AUC of the one-compartment model was below 400.

For the two-compartment PK profile, the probability of an AUC/MIC of <400 for the reduced one-compartment model was 86.3% when a regimen of 0.75 g IV infusion over 2 h every 12 h was administered and the MIC was 1 mg/L, while the probability for the two-compartment model was 36.3%. In the case of an AUC/MIC of 400 to 600, which considers efficacy and safety simultaneously, the PTA for the one-compartment model with the same regimen was 13.5%, while the PTA for the two-compartment model was 22.6%. The probability of an AUC/MIC of >600 for the one-compartment model was 0.120%, while that for the two-compartment model was 41.1% when vancomycin was administered with the same dosage regimen (Table 3).

**Figure 5:**
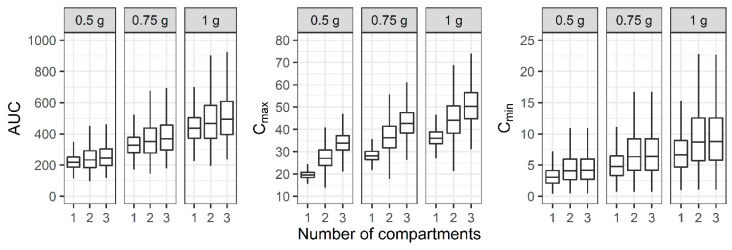
Distribution of area under the curve over 24 h (AUC), maximum concentration (C_max_), and minimum concentration (C_min_) at steady state when one-, two-, and three-compartment models were developed with three-compartment PK profile and sparse or dense sampling designs (dashed lines, typical parameter values used for simulation; horizontal line inside the box, median; ends of each box, upper and lower quartiles; whiskers, 1.5 × quartiles).

For the three-compartment PK profile, the AUC, C_max_, and C_min_ of the reduced one- and two-compartment model showed smaller values compared to the three-compartment model (Figure 5). When a regimen of 1 g IV infusion over 1 h every 12 h was administered, the median AUC of the one-, two-, and three-compartment models was between 400 and 600, while the median C_max_ demonstrated large differences between the models.

For the three-compartment PK profile, the probability of an AUC/MIC of <400 for the reduced one-, two-, and three-compartment models was 35.6, 32.7, and 25.8%, respectively, when a regimen of 1 g IV infusion over 1 h every 12 h was administered and the MIC was 1 mg/L. In the case of an AUC/MIC of 400 to 600 considering efficacy and safety simultaneously, the probability for the one-, two-, and three-compartment models with the same regimen was 58.8, 45.1, and 47.7%. The probability of an AUC/MIC of >600 for the one-, two-, and three-compartment models was 5.56, 22.2, and 26.6%, respectively, when vancomycin was administered with the same dosage regimen (Table 4).

## 4. Discussion

Although vancomycin has been used for nearly 70 years, the optimal vancomycin pharmacotherapy is still challenging. Because the therapeutic range of vancomycin is narrow and pharmacokinetics (PK) are affected by the various pathophysiology of patients, therapeutic drug monitoring (TDM) is required for personalized treatment that maximizes the efficacy and minimizes side effects [16,17]. The first consensus guideline for the therapeutic monitoring of vancomycin in adult patients advocated an AUC/MIC ≥400 as a target for the PK/PD index for serious infections with MRSA and recommended trough-only monitoring with a target of 15 to 20 mg/L as a surrogate marker for the AUC/MIC of ≥400 when the MIC is ≤1 mg/L. Multiple blood samples are required to directly calculate the AUC for a patient. Because serial sampling may not be easy in a real clinical setting, the trough concentration was recommended as a practical surrogate marker for the AUC [18]. The evidence of this recommendation was based on clinical experience, descriptive studies, and reports of expert committees rather than clinical trials. The recently revised consensus guideline for the TDM of vancomycin no longer recommends a target trough concentration of 15 to 20 mg/L for efficacy based on well-designed clinical trials [6]. This guideline advocates a personalized target of an AUC/MIC of 400 to 600 when the MIC is 1 mg/L for patients with serious MRSA infections to achieve clinical efficacy and safety. Additionally, the guideline recommends Bayesian-derived AUC-guided monitoring using a well-developed population PK model of vancomycin based on richly sampled PK data.

A population PK model is a Bayesian prior, which is used to derive an individual’s Bayesian posterior PK parameters along with information such as demographic factors, pathophysiological factors, dosing history, and drug concentration [19]. A model with good predictive performance is needed when using the model to adjust the dosage regimen and achieve precision dosing. For many people, model-informed precision dosing (MIPD) is still an unfamiliar term compared to precision medicine. According to the definition in the Precision Medicine Initiative, precision medicine is an emerging innovative approach for disease prevention, diagnosis, and treatment that takes into consideration the BSV in genes, the environment, and lifestyles [20]. With the remarkable development of genetic sequencing technology, such as next-generation sequencing, precision medicine has grown by focusing on an individual’s genetic profile rather than other environmental and pathophysiological factors [21,22]. Contrary to the traditional treatment method of taking a one-size-fits-all approach to the average patient, precision medicine is an effective method of providing tailored medicine for a specific patient group, but it has limitations in providing individualized treatment [23]. Both precision medicine and MIPD aim to give the right patient the right treatment at the right time, but precision medicine lacks the element of time. The MIPD approach incorporates the time factor into the PK model to optimize the dosage regimen of the drug selected for the patient in real-time [24]. Because of the time-varying nature of a patient’s pathophysiology, a robust PK model with good predictive performance is needed for real-time pharmacotherapy according to the PK/PD target of antibiotics [25].

To examine what a good robust model is at present when numerous PK models are being developed, we investigated the estimated PK parameters and the predicted PK/PD index of vancomycin when a model with a lesser number of compartments from a sparse sampling design was developed. In our study, when a one-compartment model was developed with the PK profile simulated with the previously reported two-compartment model, the CL was estimated to be greater than 90% compared to the parameters used in simulation, while the volume of distribution (Vd) was estimated to be less than 27% (Table 2). The importance of knowing the exact CL and volume of distribution (Vd) when administering antibiotics can be seen from the following three equations:

AUC (mg·h/L) = Dose (mg)/CL (L/h)

Maintenance dose (mg/h) = Concentration (mg/L) × CL (L/h)

Loading dose (mg) = Concentration (mg/L) × Vd (L)

If the CL is overestimated, the AUC is calculated to be smaller than the actual value, resulting in an overdose. This is also true when counting maintenance doses; if the Vd is underestimated, an insufficient loading dose will be administered. Our result pertaining to the prediction of the AUC from the PK models exhibits this risk well. Although the dosage regimen was the same, the reduced one-compartment model, in which the CL was estimated to be much larger than the CL in the two-compartment model, predicted a smaller AUC (Figure 4). Therefore, a clinician who adjusts the dose based on this prediction may attempt to administer a higher dose. The probability of meeting the PK/PD index, which is used to evaluate the appropriateness of antibiotic treatment, also showed a large difference between the developed models (Table 3). When a regimen of 1 g IV infusion over 2 h every 12 h was administered and the MIC was 1 mg/L, more than 55% of patients belonged to the PK/PD index group of AUC_<400_ for the reduced one-compartment model, while more than 57% of patients belonged to the PK/PD index group of AUC_>600_ for the two-compartment model. This result shows that a large error may occur in the prediction of the treatment effect if the blood sampling design is different in a situation where the model is made for the same patient group. This trend appeared when a model with a lesser number of compartments was established with a PK profile simulated with the previously reported three-compartment model. Although the RBias and the RRMSE of all the CL and V_SS_ were less than 15 and 35%, respectively, except that the RBias for the CL was 15.6% when the number of subjects was 12 (Table 2), it should be kept in mind that even a small difference in the estimation of the PK parameters can have a meaningful effect on the safety and efficacy of drugs with a narrow therapeutic range.

We determined the effect of the number of compartments on the prediction of the PK/PD index due to the usual limitation in the number of samples while excluding other factors as much as possible. There was no need to estimate the covariates in the stage of estimating model parameters because they were fixed at the median in the previous simulation stage. Because studies that develop a population PK model include a diverse number of subjects, we also tried to understand the effect of the number of subjects on parameter estimation. In the simulation stage, the parameters with individual differences were generated within the two standard deviations (SDs). As a result, regardless of the number of subjects, the median value of the parameter estimates of 200 datasets were almost the same. When one set is a dataset of 12 subjects, it would have been greatly affected by outliers outside of the two SDs. However, as shown in Figure 2 and Figure 3, even if it is generated within the two SDs, when the number of subjects is small, it is highly likely that the parameter estimate is different from the value used for simulation. The accuracy and precision of the population PK/PD parameter estimates are affected by various factors, including the sample size of patients, the number of measurements per patient, sparse or intensive sampling design, the degree of the BSV, and the magnitude of the within-subject variability (WSV) [5,26]. For a one-compartment model with first-order absorption, the sample size required to estimate the absorption rate within a precision level of 20% for a 95% confidence interval and a power of 90% was 30 for a sparse three-sampling scheme and 20 for a dense six-sampling scheme [27]. When testing a hypothesis using a one-compartment PK model, the sample size increases from 28 to 416 as the BSV increases from 20 to 80% for a power of 80%, mean difference of 20%, and WSV of 10% [28]. Although covariates were excluded from the analysis in this study, it should be noted that a larger number of subjects are required for studies that include covariates [29].

This study had some limitations. First, we assumed that the two previously reported PK models of vancomycin were the models with a good predictive performance. However, it cannot be ruled out that at least one of the two is not a good model. Second, only two arbitrarily selected PK models were evaluated in this study. Although we could not examine all PK models of vancomycin, we carefully chose two robust models and quantitatively assessed the importance of developing good models in antibiotic therapy. Third, we arbitrarily selected the sampling time. Although it was not possible to investigate all possible conditions, it must be important to note that a model made with a sparse sample can make a big difference in predicting the treatment effect. Fourth, we fixed the covariate values in our simulation, which were included in the reported models. However, because every patient is assumed to have the same covariate and the process of simulating and finding the covariate is omitted, we were able to clearly compare the bias and precision of the parameter estimates without other confounding factors.

In conclusion, estimated PK parameters and a predicted PK/PD index from models built with sparse sampling designs that cannot support the actual PK profile can be significantly inaccurate and unprecise. This might lead to a misprediction of the PTA and the selection of improper dosage regimens when clinicians prescribe antibiotics. A developed model should be continuously improved through the PK/PD data collection and repeated external validation must be performed to be of practical help in patient treatment.

## Figures and Tables

**Table 1:** Population PK parameter values used for simulation.

Parameter	Estimate	BSV
Two-compartment model [12]		
$\mathrm{CL}\;{=\theta }_{1}{\;\times \;(1+\theta }_{2}\;\times \;\left(\mathrm{CLCR}\;-\;83\right))$		
θ_1_ (L/h)	2.83	77%
θ_2_	0.0154	
$V1\;{=\theta }_{3}{\;\times \;(1+\theta }_{4}\;\times \;\left(\mathrm{BW}\;-\;94.5\right))$		
θ_3_ (L)	24.2	34%
θ_4_	0.00638	
Q1 (L/h)	11.2	
$V2\;{=\theta }_{5}{\;\times \;(1+\theta }_{6}\;\times \;\left(\mathrm{BW}\;-\;94.5\right))$		
θ_5_ (L)	32.3	
θ_6_	0.0169	
Residual proportional error	8.19%	
Three-compartment model [13]		
$\mathrm{CL}\;{=\theta }_{7}{\;\times \;(1+\theta }_{8}\;\times \;\left(\mathrm{CE}\;-\;56.75\right))$		
θ_7_ (L/h)	4.01	33.9%
θ_8_	0.00752	
V1 (L)	8.01	27.9%
Q2 (L/h)	4.95	
V2 (L)	15.4	34.3%
Q3 (L/h)	9.09	
V3 (L)	6.21	56.9%
Residual proportional error	6.64%	

BSV, between-subject variability; CL, total clearance; V1, central volume of distribution; V2, volume of distribution for the first peripheral compartment; Q1, intercompartmental clearance between V1 and V2; V3, volume of distribution for the second peripheral compartment; Q2, intercompartmental clearance between V1 and V3; CLCR, creatinine clearance; CE, glomerular filtration rate estimated by CKD-EPI equation.

**Table 2:** Relative bias (RBias) and relative root mean square error (RRMSE) of total clearance and steady-state volume of distribution when the number of compartments for PK model is equal to or less than that for the initially developed model.

Number of Compartments		Subject Number	CL		V_SS_	
Simulation	Estimation		RBias (%)	RRMSE (%)	RBias (%)	RRMSE (%)
2	1	12	90.1	92.2	−28.5	28.7
		25	92.0	93.2	−28.1	28.2
		50	92.6	93.1	−27.7	27.8
		100	91.4	91.7	−27.9	27.9
	2	12	−2.30	35.1	1.87	14.9
		25	3.10	25.8	0.053	10.2
		50	3.02	17.7	0.661	6.93
		100	1.96	13.0	0.441	5.27
3	1	12	15.6	18.3	−7.94	9.31
		25	14.0	15.4	−8.59	9.17
		50	14.3	15.0	−8.55	8.88
		100	14.5	14.8	−8.40	8.59
	2	12	7.79	13.0	13.2	15.8
		25	6.35	9.62	13.5	14.7
		50	6.98	8.61	13.1	13.7
		100	7.27	8.03	13.3	13.6
	3	12	1.81	9.66	1.44	7.75
		25	0.395	6.42	0.684	4.75
		50	0.715	4.64	0.327	3.51
		100	0.929	3.34	0.325	2.49

CL, total clearance; V_SS_, steady-state volume of distribution.

**Table 3:** Probability (%) for three PK/PD parameters of vancomycin when one- and two-compartment models were developed with 10,000 two-compartment PK profiles and sparse or dense sampling designs.

Dose	PK/PD Index	1 Compartment	2 Compartments
0.5 g	AUC_<400_	99.9	59.0
	AUC_400–600_	0.120	20.2
	AUC_>600_	0.000	20.8
0.75 g	AUC_<400_	86.3	36.3
	AUC_400–600_	13.5	22.6
	AUC_>600_	0.120	41.1
1 g	AUC_<400_	55.2	22.6
	AUC_400–600_	39.4	19.6
	AUC_>600_	5.42	57.8

AUC_<400_, AUC/MIC of <400; AUC_400–600,_ AUC/MIC of 400 to 600; AUC_>600_, AUC/MIC of >600.

**Table 4:** Probability of target attainment (%) for three PK/PD indices of vancomycin when one-, two-, and three-compartment models were developed with 10,000 three-compartment PK profile and sparse or dense sampling designs.

Dose	PK/PD Index	1 Compartment	2 Compartments	3 Compartments
0.5 g	AUC_<400_	100	96.5	95.5
	AUC_400–600_	0.000	3.50	4.50
	AUC_>600_	0.000	0.000	0.000
0.75 g	AUC_<400_	83.0	65.0	59.1
	AUC_400–600_	17.0	31.5	36.4
	AUC_>600_	0.000	3.51	4.54
1 g	AUC_<400_	35.6	32.7	25.8
	AUC_400–600_	58.8	45.1	47.7
	AUC_>600_	5.56	22.2	26.6

AUC_<400_, AUC/MIC of <400; AUC_400–600,_ AUC/MIC of 400 to 600; AUC_>600_, AUC/MIC of >600.

## Data Availability

The datasets generated and/or analyzed during the current study are available from the corresponding author on reasonable request.
